# Relaxation Behavior of Cerclage Cables and Its Effect on Bone Clamping Force

**DOI:** 10.3390/bioengineering11121289

**Published:** 2024-12-19

**Authors:** Audrey Moffat, Wonsuk Kim, Tahsin Rahman, Kayla Podlewski, Craig Silverton, Alan Argento

**Affiliations:** 1 Department of Mechanical Engineering, University of Michigan-Dearborn, Dearborn, MI 48128, USA; aemoffat@umich.edu (A.M.); wskim@umich.edu (W.K.); kpod@umich.edu (K.P.); 2 Department of Orthopaedic Surgery, Henry Ford Health System, Detroit, MI 48202, USAcsilver1@hfhs.org (C.S.)

**Keywords:** cerclage, relaxation, bone, fixation

## Abstract

Cerclage is an orthopedic surgical fixation technique using a cable wrapped, tensioned, and secured around a bone’s circumference. It is important to minimize the loss in cable tension that often occurs due to stress relaxation. The purpose of this work was to study the effect of tensioning protocols on the long-term loss of tension due to stress relaxation. The native mechanical properties and relaxation behavior of the cables were determined using traditional mechanical testing machines and methods. Four step-wise cable tensioning protocols were then trialed to compare the cable tension losses. A testing apparatus was developed to simultaneously measure cable tension and the resulting clamping force on a real bone. A five-parameter linear viscoelastic model was used to fit relaxation data to estimate the long-term relaxation of the cables beyond the time of the experiment. The four cables were found to have similar mechanical and viscoelastic behaviors. A two-step cable-tightening protocol was found to significantly reduce cable tension loss when compared to a one-step protocol for all cables. The benefit of the two-step protocol was reinforced by the relaxation results of the cable wrapped and tightened around a pig femoral bone. These results indicate that one retightening step should be conducted during the surgical placement of a cerclage cable to reduce the loss of cable tension resulting from relaxation.

## 1. Introduction

Cerclage cables are commonly used for fracture fixation, most notably for periprosthetic fractures, among many other applications [1,2,3,4]. During the surgery, the surgeon wraps and tensions cables around the bone’s circumference. The attendant pressure applied to the bone closes and stabilizes the fracture so the bone can subsequently heal. Cerclage is often used to repair periprosthetic femur fractures around a total hip arthroplasty [5,6] as it allows for the apposition of fracture fragments otherwise precluded from bicortical screw placement due to arthroplasty components being in the way. This technique has demonstrated improved outcomes [7], better stability, and a shortened return to weight bearing [8]. These issues are of particular significance for fractures in elderly patients [9]. Cerclage has been found to be primarily useful for spiral and oblique fractures due to its ability to resist shear loads in those cases [4].

Though cerclage is an accepted fixation method, there are some issues with its use. Ex vivo experiments of the integrity of cerclage fixation of the human femoral bone during the application of loads showed inhomogeneous interface pressure distribution [10] partly due to the cable spanning some regions of the cross-section without contact. This is thought to reduce the chance of loss of blood supply to the bone; however, the danger of necrosis due to overly tight cerclage [11] remains a concern as specific techniques have been developed to prevent this [12]. Additionally, due to the surgeon’s uncertainty of both the actual contact pressure during cerclage and the optimum fixation system [5] for a specific surgery, there are no definitively established measures of what tension should be used for bones of different sizes and ages, as well as for the bones of people suffering from diseases such as osteoporosis. There are also no medical standards for the initial cable installation tension, the tightening rate, and the need for retightening, resulting in variations of tightening protocols among surgeons. Insufficient tension can result in cable loosening and poor healing, and methods to increase cable tension are being actively studied [13,14]. In general, the integrity of mechanical fixation is known to be of paramount importance [15,16] to bone healing quality. However, the relationship is complicated, with poorer healing quality also resulting from overly rigid fixation [17]. This point is particularly relevant to cerclage where the pressure distribution varies around the bone and within the fracture contact surfaces, and the uncertainty of the relationship between cable tension and these pressures can result in regions with pressure spikes.

Following tightening of the cerclage cable during surgery, the cable often experiences loosening due to cable stress relaxation and imperfect crimping of the crimp nut. In a study focused on the initial cable loosening of five cable systems, all were found to experience tension loss, with the tension reduction ranging from 20–50% following the initial cable fixation and tensioner removal [18]. The mechanical properties of various metal alloy and nylon cerclage cables were compared in [19], showing early loosening in stainless-steel and cobalt-chrome cables. However, in a study of double-looped cerclage with a crimp, no loosening or failure due to the crimp was observed [14]. Tension loss in wire and cable cerclages were compared [20]. It was found in cyclic mechanical testing that crimped cerclage cables provided the least amount of tension loss compared to twisted cerclage wires. Metallic and non-metallic cables were compared in models with and without tissue to assess stability and compressive forces [21]. The results showed that metallic cables posed a higher risk due to sharp edges and wear, potentially damaging surrounding tissue and loosening over time. In contrast, non-metallic cables offered comparable stability with lower compressive forces and higher elasticity, making them less likely to harm biological tissues. In a study mimicking cerclage fixation and walking motions, tension loss was seen in all four tested cerclage cables tightened around a cadaver femur [22].

The focus of this work was the study and minimization of long-term cable stress relaxation. Two common cerclage cables in two sizes were used in the study. First, traditional stress–strain and viscoelastic tests were conducted to determine, respectively, the failure stresses and native relaxation behaviors of the cables. Then, four cable-tightening protocols were developed and compared relative to cable tension loss due to cable stress relaxation. The initial drop in stress that sometimes occurs during surgery due to the application of insufficient pressure to the crimp nut after cable tightening is not a relaxation process and was not part of this study. The results show that the four cables have similar mechanical and viscoelastic behaviors. A two-step tightening routine was found to greatly reduce cable relaxation with only a small increase in complexity compared to a one-step routine. A multi-step routine further reduced relaxation relative to the two-step protocol, but the slight improvement in relaxation is not commensurate with the added surgical complexity the multi-step protocol would demand. An experiment using a cable wrapped around a pig femur showed that cable relaxation can reduce the net clamping force applied to a bone to close a fracture line. Curve fits of the measured data were used to calculate estimates of cable tension after relaxation for 1 month.

## 2. Materials and Methods

### 2.1. Cerclage Cables

Four cerclage cable types manufactured by Stryker Corporation (Kalamazoo, MI, USA) were used for all testing. These are made from stainless steel and vitallium (a CoCr alloy) materials, each having d = 1.6 mm and 2.0 mm where d denotes the diameters of cables. All cable experiments were conducted at an ambient room temperature of 22 °C.

### 2.2. Stress–Strain Tests

Stress–strain tests were conducted on the four cable types to ensure that the stresses that developed in the subsequent cable-loading procedure tests were well below the failure stresses of the cables, measured at the same testing rate. The cables were cut into 4-inch-long pieces for the stress–strain tests. An Instron (Norwood, MA, USA) series 6800 Universal Testing machine was used with a 5 kN load cell and adapter. The 4-inch cable test specimen was secured within the center of the testing grips, and the initial grip length (specimen gauge length) was set at 67.47 ± 5.90 mm using a caliper. Tension was then applied to the specimen at a rate of 12 mm/min until failure. This rate was chosen based on trials of measurements of the average speed at which two orthopedic surgeons tensioned cerclage cables around a sawbone [23] using a typical surgical tensioner. Each surgeon conducted two trials. The overall range was 9.9 mm/min–12.5 mm/min, with an average of 11.4 mm/min. For all experiments, the average rate was rounded up to 12 mm/min. Engineering stress and strain were calculated from the load–displacement data using the conventional axial loading expressions:

(1) $$\sigma =\frac{F}{A_{o}},\;\;\varepsilon =\frac{∆l}{l_{o}},$$

where σ is the engineering axial normal stress, F is the current applied force, ${\;A}_{o}$ is the original cross-sectional area of the cable, ε is the linear engineering normal strain,${\;l}_{o}$ is the original gauge length, and l is the current gauge length during the test so that ∆l = l − ${\;l}_{o}$ is the specimen’s current elongation.

Each reported graph is the result of an average of tests on two different samples. The reported Young’s Modulus is based on a linear fit of the initial 0.2% of the strain of the average curves. The maximum stress is the average of the maximum stresses the two curves reach. The failure strain is the average of the strains of the two curves at complete failure. The standard deviation is reported for each property.

### 2.3. Cable Relaxation Tests

In these tests, the conventional relaxation behavior of the four cables was compared. A TestResources (Shakopee, MN, USA) 100-Q-225-6 loading machine with a 1.1 kN load cell was used for all relaxation tests. Each cable was positioned within the grips of the loading machine with a gauge length of 36.14 ± 2.11 mm. All relaxation tests were conducted using a displacement-controlled mode. The test had two consecutive segments. Segment A was programmed to stretch the sample at a rate of 100 mm/min until a force of 400 N was reached. This high rate provides a near step input of force and serves to minimize viscoelastic dissipation during the input phase of the experiment and is the conventional technique to determine the native viscoelastic material behavior from Segment B [24]. The displacement at the maximum load depends on the cable diameter and its specific stiffness and so varies from cable to cable. When Segment A ended, Segment B immediately began. During Segment B, the specimen stretch was held constant for 20 min and the reduction in tensile force in the specimen was measured. The initial maximum 400 N force input was chosen based on the cable manufacturer’s recommendation, as well as preliminary laboratory testing in which a cable was stretched by a surgical tensioner against a load cell in the test machine. This force was achievable by the tensioner and is approximately 73% of the lowest maximum tension (550 N) recommended by the manufacturer among the four cable types. Load data were normalized by the maximum force at the end of Segment A.

In preparation for the curve fitting of Segment B, the Segment A test data were trimmed and Segment B was shifted to start at t = 0. Curve fits of Segment B were determined using the well-known 5-parameter linear viscoelastic model [24]:

(2) $$F(t)=F_{1}+\sum_{i=2}^{3}F_{i}e^{-\frac{t}{\tau_{i}}}$$

where F(t) is the curve-fitting force during relaxation as a function of time t, $F_{1}$ is the equilibrium force when fully relaxed (t=∞), $F_{2}$ and $F_{3}$ are parameters corresponding to the forces of the model’s two viscoelastic branches at t=0, and $\tau_{2}$ and $\tau_{3}$ are the relaxation times. The five constants were determined by curve fitting of the measured force relaxation data using the method of nonlinear least squares with the Levenberg–Marquardt algorithm [25] in MATLAB (version R2021b, MathWorks Corporation, Natick, MA, USA). Because a series of curve fits can yield multiple combinations of parameters that provide a reasonable fit of the data, here, a strategy of carefully selecting the starting values for the Levenberg–Marquardt curve-fitting routine based on known characteristics of the relaxation behavior was used. Specifically, the starting value of $F_{1}\;$ was selected to be the measured force $\left(F_{f}\right)$ at the end of Segment B ($t=t_{f}$); $\left(F_{max}-F_{f}\right)/2\;$ was used for the starting value of both $F_{2}$ and $F_{3}$, where $F_{max}$ is the measured maximum force at t=0; the starting value of $\tau_{2}$ was assumed to be a short time of 1 s; the starting value of the long-term relaxation time $\tau_{3}$ was set to be $t_{f}/2$; and all five parameters were constrained to be greater than 0. The MATLAB algorithm tends to determine a local solution in the vicinity of the starting values. To determine the resulting parameter values that yield the best solution, the relaxation data were re-fitted after iteratively changing the starting values. The iteration strategy used was to vary $F_{1},\;F_{2},\;$ and $F_{3}$ one by one from very small values of 1% of $F_{i}$’s starting values selected above to larger values that are three times the starting values. $\tau_{2\;}and$ $\tau_{3}$ were also varied one by one from 1% of the starting values to $t_{f}$ and ${2t}_{f}$, respectively. The quality of the curve fit after each parameter iteration was assessed using the adjusted R^2^ value provided by MATLAB.

### 2.4. Cable Loading Procedures

Repetitive cable loading–relaxation tests were designed to test relaxation resulting from different procedures of cerclage cable retightening. Four loading procedures were tested, each consisting of pairs of input–output segments (like Segments A and B described above). The number of input–output pairs and their durations varied from case to case to study the effect on cable tension loss due to relaxation. Each load input segment was programmed to run at 12 mm/min, which corresponds to the average rate of loading measured in trial experiments with a cerclage surgical tensioning tool, as described in Section 2.2. Once the desired load was achieved, the relaxation segment began and continued for a specified time with the specimen stretch held constant. In each procedure described below, a series of load–relaxation segments were conducted, with only a brief pause after each relaxation phase to save retrieved data. All four procedures required roughly 12.5 min to complete, not including the short times the loads were applied. For these experiments, a maximum input load of 440 N was used. This load challenges the cables slightly more than the 400 N load used in the conventional relaxation experiments but is still less than the manufacturer’s recommended maximum. The specific relaxation procedures are listed below.

1. One-step procedure. This mimics a cerclage cable attachment process in which the surgeon tightens the cable only once during the surgery. Here, the initial load input (i.e., cable tension) of 440 N was followed by a 12.5 min relaxation period.
2. Two-step procedure. This mimics an approach where the cable is tightened when the cable is first wrapped around the bone and one additional time after the initial cable tightening. Specifically, the initial load input of 440 N was followed by a 6.25 min relaxation period, then a second tightening was conducted to return the tension back to 440 N, followed by a relaxation period of 6.25 min.

In the next two procedures, the load is increased to 440 N in 5 steps with relaxation periods after each step.

3. Five-step-1 procedure. Here, the initial load was 320 N followed by a 150 s relaxation period. Then, the load/relaxation pattern was repeated with loads increased to 360 N, 400 N, 440 N, and 440 N, each followed by a 150 s relaxation period.
4. Five-step-2 procedure. This procedure had the exact same loading pattern as Five-step-1, but the relaxation periods were all 100 s, except for the last one, which was 350 s.

### 2.5. Cable Relaxation When Applied to a Pig Femur

**Figure 1:**
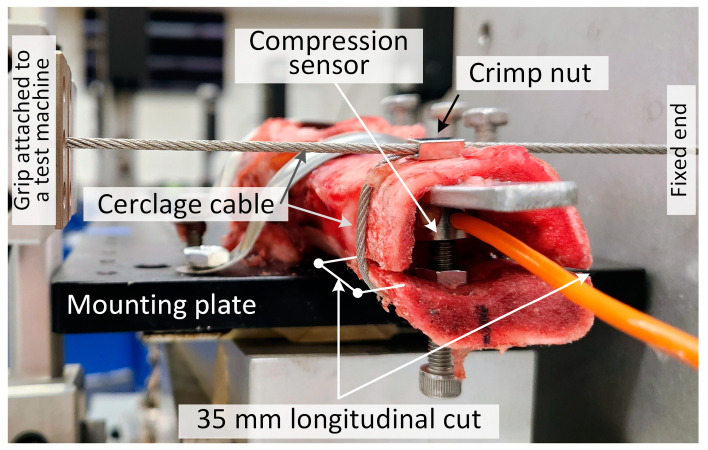
Experimental setup: stainless-steel cerclage cable wrapped around a pig femur.

Here, the relaxation behavior of the stainless-steel 2.0 mm diameter cable was studied when wrapped around a pig femoral bone. The goal was to confirm the occurrence of relaxation of the cable tension and that of the net clamping force applied to the bone when the cable is wrapped around a real bone. The pig femur was obtained from a local slaughterhouse (Scholl’s, Blissfield, MI, USA), transported on ice, and frozen at −20 °C within 4 h of slaughter. The experiment occurred about 1 month after freezing the bone. The bone was thawed and cleaned of excess meat and fat, and the joint at one end was cut off. The bone in the experimental apparatus is shown in Figure 1. A 35 mm longitudinal cut was made at the end of the bone using a jigsaw with a 1.7 mm thick blade. The final dimensions of the tested bone were 120 mm long with outer and inner diameters of approximately 29 and 20 mm, respectively, at the location of the cable. The bone was clamped to a steel mounting plate at the uncut joint end and the cerclage cable wrapped around the region of the bone with the longitudinal cut. The TestResources machine described above was used to apply tension to the cable. One end of the cable was inserted into the grips of the machine, and then the cable was wrapped around the pig femur and passed through an un-crimped Stryker crimp nut. The other end of the cable was secured. The net clamping force due to the action of the cerclage cable on the bone was measured using a small compression sensor (ATO Micro 100 kg Compression Load Cell) inserted inside the bone near the cerclage cable. This force sensor bears against a metal plate at one end and a ¼-20 inch screw passed through a 3/16-inch hole drilled in the bone. Before the test, the screw was adjusted using nuts inside and outside the bone’s lumen to just make contact with the sensor. The pair of nuts also locked the screw in place. During the experiment, the test machine applied tension to the cable using the two-step cable-loading procedure described above. The results are given for the cable tension measured by the TestResources force sensor and the simultaneous compressive clamping force measured by the ATO sensor within the bone.

### 2.6. Statistics

Statistical and numerical calculations were conducted using Excel (Microsoft Corporation, Redmond, WA, USA). Unpaired, two-tailed, t-tests were performed at the 5% level of significance to evaluate the statistical differences among cables and loading procedures. For the comparison between the one-step and two-step procedures, effect sizes based on Hedge’s g with bias correction were also determined.

## 3. Results

Note that in all graphs, the abbreviations ss1.6 and ss2 denote stainless-steel cables of 1.6 and 2.0 mm diameters, respectively, while vit1.6 and vit2 denote vitallium cables of 1.6 and 2.0 mm diameters, respectively.

### 3.1. Stress-Strain

**Figure 2:**
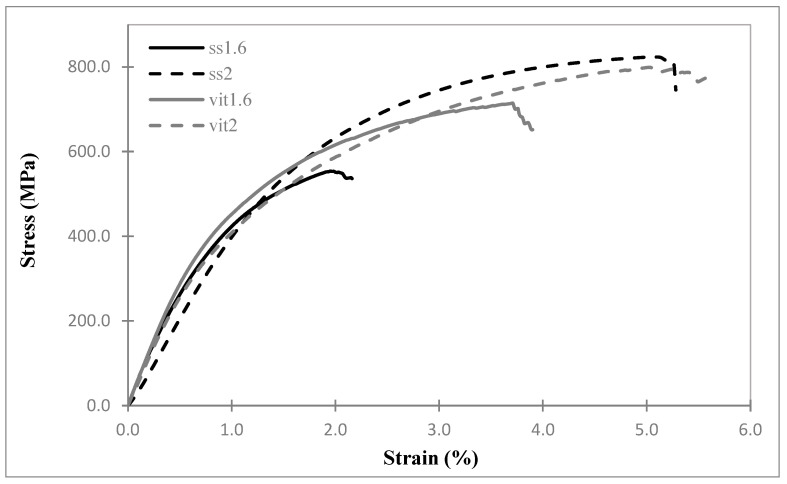
Engineering stress–strain curves for four cable types tested at a loading rate of 12 mm/min.

The engineering stress–strain curves for four variations of the cerclage cable are given in Figure 2 while material properties extracted from the data (Young’s modulus, maximum stress, and failure strain) are given in Table 1. The smallest failure strain and maximum stress occurred in ss1.6, while ss2 had the largest maximum stress and the smallest modulus. The vit1.6 cable had the largest modulus and vit2 had the largest failure strain. The four cables all show a progressive nonlinear stress–strain shape, and the maximum stress occurs nearly at the failure strain in all four cables.

### 3.2. Cable Relaxation Behavior

**Figure 3:**
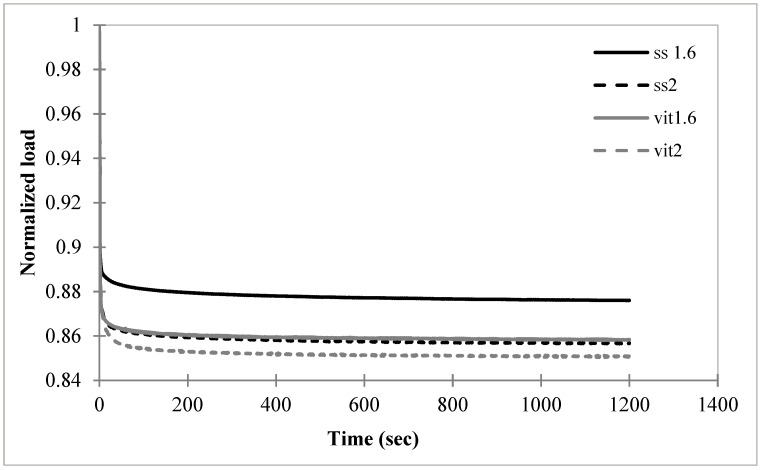
Average normalized load (i.e., cable tension) versus time relation curves for four surgical cable types (*n* = 3) with input tension applied at a rate of 100 mm/min until a load of 400 N was achieved.

The results of the force–relaxation tests for the four variations of cerclage cables are given in Figure 3. All cables are seen to immediately undergo a pronounced loss of tension followed by a gradual relaxation. Cable ss1.6 had the least amount of relaxation with a loss of 12.40% of the initial tension. Cables vit1.6, ss2, and vit2 had similar amounts of tension loss due to relaxation, specifically 14.16%, 14.32%, and 14.89%, respectively.

Statistical comparisons of the normalized tension values at t = 1200 s are given in Table 2. The differences in tension loss due to relaxation between the four cables were found to be insignificant, *p* > 0.05.

### 3.3. Effects of Cable Loading Procedures

**Figure 4:**
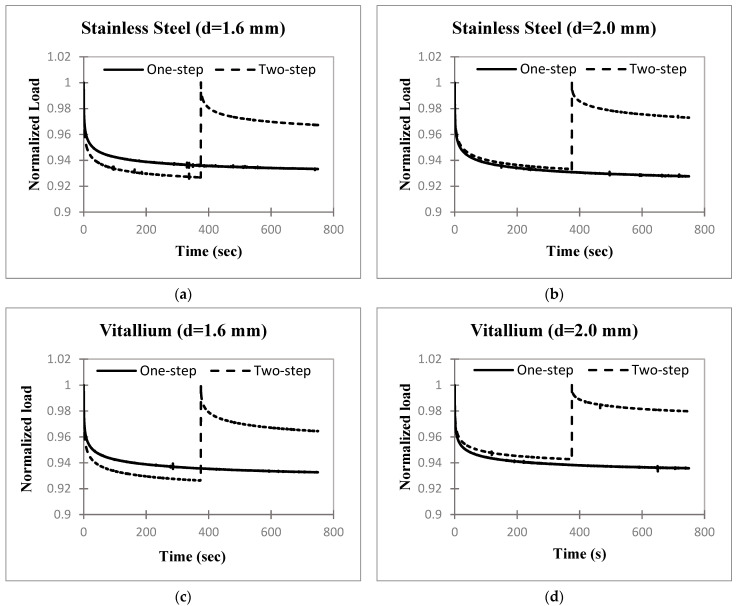
Average normalized load (i.e., cable tension) versus time (*n* = 4) for the one-step and two-step cable-loading procedures of the four cable types: (**a**) stainless steel 1.6 mm; (**b**) stainless steel 2.0 mm; (**c**) vitallium 1.6 mm; (**d**) vitallium 2.0 mm.

**Figure 5:**
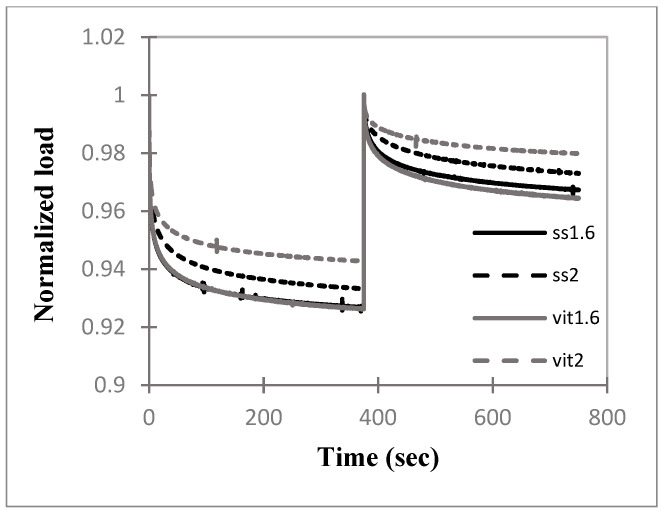
Average normalized load (i.e., cable tension) versus time (*n* = 4) of the four cable types for the two-step cable loading procedure.

The results for the one-step and two-step cable loading–relaxation procedures for the four cables are shown in Figure 4. For easier comparison, these are also plotted together in Figure 5 for the two-step case. Reloading the midway during the relaxation period in the two-step procedure resulted in reduced cable tension loss for all four cables. The cable tensions from the one-step and two-step procedures were compared at t = 750 s in Table 3. For all four cables, there was a significant difference between the one-step and two-step cable tensions at t = 750 s as seen in both *p*-values and effect sizes. The lower portion of the table shows a comparison of the vitallium and stainless-steel cables of the same size for the two-step procedure at 750 s. The difference between ss1.6 and vit1.6 tensions was statistically insignificant (*p* = 0.3212 > 0.05), while the difference between ss2 and vit2 tensions was statistically significant (*p* = 3.45 × 10^−2^ < 0.05).

**Figure 6:**
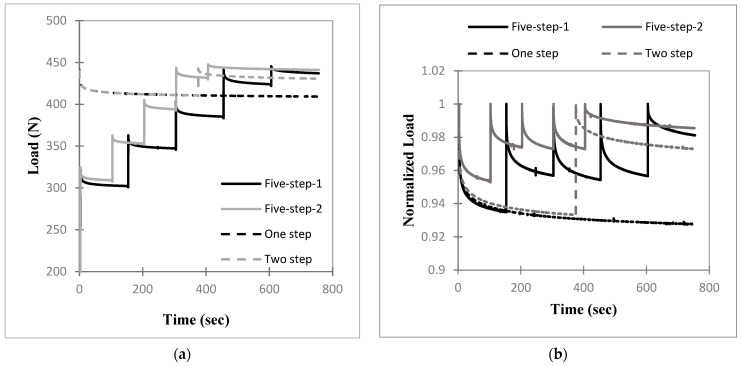
Four repeated relaxation protocols using the 2.0 mm diameter stainless-steel cable. (**a**) Non-normalized load versus time; (**b**) normalized load versus time.

The five-step procedures were studied using cable ss2. These procedures are given in Figure 6 alongside the one- and two-step procedures. To more easily visualize the five-step procedures, the results are presented in non-normalized form in Figure 6a and in normalized form in Figure 6b. At 750 s, the five-step-1 and 2 procedures had tension losses of 2.15 and 1.85%, respectively, while the one- and two-step values were 7.22 and 2.70%, respectively.

Of the four relaxation procedures tested, five-step-2 had the best preservation of cable tension, although procedure five-step-1 had only a 0.30% point greater loss of tension. However, these relaxation procedures were carried out by means of the gradual addition of force over many inputs, which would result in increased surgical complexity compared to the one-step and two-step procedures.

The long-term relaxation behavior of the cables was estimated using the five-parameter model, Equation (2), for the one-step and two-step cases of the 2.0 mm stainless-steel cable given in Figure 4. Note that the second relaxation phase of the two-step case was fit. Almost all iterated solutions resulting from the approximately 17,000 tested combinations of the starting parameter values had adjusted R^2^ values of about 0.99 and all yielded the same set of parameters within ±0.25%, except a solution with a slightly lower R^2^ value. Every combination that showed fits with R^2^ values lower than 0.98 yielded solutions with one relaxation time of essentially zero, which reduces the model to a three-parameter model. Such solutions were rejected. In every case, the selected curve fit for the approximation of the data was the one with the highest R^2^ value. Table 4 gives the cable tensions calculated from the best curve-fitting function at 1 day, 1 week, and 1 month. The results show that in both the one-step and two-step cases, the cables relax negligibly after 750 s, about 0.11% at 1 month.

### 3.4. Cable Relaxation When Applied to a Pig Bone

**Figure 7:**
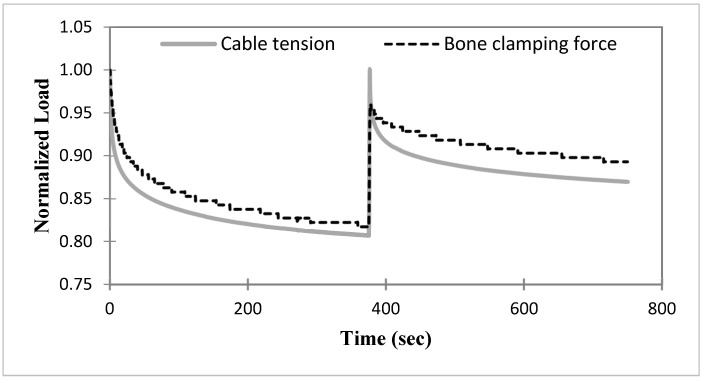
Average (*n* = 2) normalized loads (i.e., cable tension and bone clamping force) versus time of the ss2 cable, wrapped around a bone, for the two-step loading procedure.

The results for the two-step loading-relaxation tests of stainless-steel cables (d = 2.0 mm) wrapped around a pig femur are shown in Figure 7. Solid and dashed curves denote cable tension and clamping force, respectively, obtained by averaging results from two tests. The cable tension and clamping force were normalized by their respective maximum forces of 440 and 199 N, respectively. A very similar relaxation trend was seen in both the cable tension and bone clamping force. At 750 s, the losses of the cable tension and clamping force were 13.0% and 10.7%, respectively.

The five-parameter model, Equation (2), was fit to the second relaxation phases of the bone clamping force and the cable tension using the iteration strategy described previously. The resulting curve fits had very high adjusted R^2^ values and variant solutions for lower R^2^ values, as in the previous cases. Table 4 gives the bone clamping force and cerclage cable tensions at 1 day, 1 week, and 1 month calculated using the best-fit model, R^2^ ≥ 0.99. Both the clamping force and cable tension in the bone experiment are seen to have small relaxation at times beyond 750 s of about 1.91% and 0.35% at 1 month, respectively.

## 4. Discussion

Metallic cerclage cables are complex, manufactured structures, not monofilament metal wire [26]. Specifically, they are multifilament systems consisting of strands of monofilaments wound into bundles, with these bundles wound together to form the cables [27]. The exact winding pattern usually depends on the diameter of the cable. Because of the structural nature of the cables, the mechanical test results would not typically be the same as those of the same monofilament material [28]. This can be seen by comparing the cable moduli in Table 1 to the average Young’s modulus of three common stainless-steel alloys (207 GPa [29]) and that of Vitallium (about 225 GPa [30]). This is also reflected in the differences between the stress–strain curves for the same cables of different sizes in Figure 2, which would be nearly identical for monofilament material specimens having different diameters. Another consequence of the structural nature of a cerclage cable is that the measured relaxation is likely the result of the inherent viscoelasticity of the cable material as well as structural aspects of the cable such as friction between the strands, the tightness of the winding, and the coil angle.

The key parameter of interest from the mechanical test results in Table 1 is the maximum stress value for each cable. This is the peak of each stress–strain curve (Figure 2) and serves as a check that the stresses induced in each cable during the cable-loading experiments did not approach the maximum stress of that cable. The maximum stress is notably lower in the 1.6 mm stainless-steel cable than the others. The maximum stress that developed in that cable in the relaxation experiment of Figure 4 is about 219 MPa, which is less than half the maximum stress in Figure 2. This indicates that the onset of failure processes did not occur in any of the relaxation experiments and so did not influence the results for any of the cables. In the stress–strain tests of Figure 2, only two cable samples were tested for each cable type. However, the failure stresses are more than double the cable loading stresses even considering the standard deviations. Given that no statistical calculations were conducted based on these stress–strain tests, two trials are considered adequate to provide an estimate of the failure stresses.

The relaxation experiment that produced Figure 3 used a very high rate load input, which is the standard method for extracting the viscoelastic relaxation properties from a material [24]. The cable tension losses in Figure 3 at 1200 s, compared to the initial tensions, ranged among the cables from 12.4% to 14.9%; however, these differences were found to be insignificant in Table 2. Thus, the four cables performed in a statistically identical manner in this experiment.

The load input rate used in Figure 4, Figure 5, Figure 6 and Figure 7 was based on the cerclage surgery rate measured in loading trials using a cerclage surgical tensioning tool by two orthopedic surgeons. The measured relaxation responses in Figure 4, Figure 5, Figure 6 and Figure 7 therefore show the tension loss by the cables during experiments that model surgical loading rates. The tension losses in Figure 4, Figure 5 and Figure 6 are lower than those in the experiment of Figure 3 because some relaxation occurred during the lower rate input phase of Figure 4, Figure 5 and Figure 6. Significant differences in cable tension loss between the one-step and two-step loading procedures were measured in all four cable types (Table 3). These results indicate that one retightening step should be conducted during cerclage surgery to reduce the loss of cable tension resulting from the mechanical relaxation of the cables. In Table 3, the two-step experiment tension loss of the 2.0 mm stainless-steel cable was significantly greater than that of the 2.0 mm vitallium cable. This did not occur for the 1.6 mm cables; however, the final load values in the 2.0 mm cables differ by only 0.7%, which is not relevant from a practical standpoint.

Of the four relaxation procedures tested, five-step-2 had the best preservation of cable tension, although this was only slightly better than that of five-step-1. These relaxation patterns were carried out by means of the gradual addition of force over many inputs, which requires a more laborious process.

Surgical efficiency remains paramount for clinical decision making. While sequential tightening can result in marginal improvement in final compressive force, it is not pragmatic to undergo several tightening steps, for example, more than three steps. Given that the five-step-2 procedure for the 2.0 mm stainless-steel cable resulted in only a 0.85% improvement in tension preservation compared to the two-step procedure of the same cable, the added surgical complexity of the five-step procedure precludes its use over the two-step procedure. Based on these results, we therefore recommend two tightening steps, which would allow for the surgeon to proceed with initial tightening, redirect their attention to another cable or surgical step such as wound cleansing, and then return a few minutes later for final tightening and crimping.

The five-step-2 procedure in Figure 6 was found to minimize tension loss among all the protocols but is likely too cumbersome for a typical surgery. However, the results from this protocol may indicate that a very slow, continuous tightening process over a 400 s time period may similarly preserve cable tension, and the procedure may be feasible in actual surgery. Longer time periods are expected to further preserve cable tension.

For each of the four cable types in Figure 4, the normalized force values for the one- and two-step cases at t = 375 s (the time at which retightening occurs) differed by at most 1.0% (in the vit1.6 case) prior to retightening. Since the two experiments are identical up to that point, the two values should be equal. The very small differences are likely due to slight variations in the cables and slight, unpredictable differences between the conduction of the experiments, which are unavoidable.

The experiment in Figure 1 demonstrates a method to determine the net dynamic clamping force acting across a bone during a cerclage procedure. The method does not rely on the use of contact pressure sensors, which may produce pressure values that drift over time, precluding their use in relaxation and other time-dependent experiments. Although based on only two experiments, the relaxation results for the cerclage cable wrapped around the bone in Figure 7 demonstrate a loss of cable tension and net clamping force in a real bone due to relaxation. The two trials also show the potential for mitigating the loss of net clamping force through a two-step loading procedure. The tension loss of the cerclage cable wrapped around the bone at 750 s in Figure 7 was 13.0%, which is much larger than the tension loss of 2.7% measured in Figure 4 (ss2) from the cable-only test. It is thought that additional sources of viscosity in the cable/bone fixation system played significant roles in the tension loss during the relaxation test of Figure 7. These could be due to the cable’s interaction with the bone, which results in friction forces and normal contact forces applied to the cable that do not occur in the mechanical test of Figure 4. Additionally, when wrapped around the bone, in addition to axial tension, as in the mechanical test, the cable is also subjected to bending and possibly shear, which might increase the relaxation.

The use of the pig femur is a limitation in the experiment in Figure 1. It has been found that animal femurs do not generally match the biomechanical and morphological characteristics of the human femur [31]. However, the pig femur is widely used in biomechanical bone experiments [32] prior to advancement to human femurs due to its low cost and wide availability. The human femur tends to have larger cortical thickness and bending stiffness than the pig femur, but human and pig femurs are similar in density [33] and medullary diameter [31]. The porcine femur is assumed to be adequate for the experiment in Figure 1 given that its purpose was only to demonstrate a method to determine net clamping force and confirm its reduction in that force in concordance with the cable force relaxation. A critical factor necessitating the use of human femurs for detailed, wide-scale statistical studies of the effect of cerclage on fixation is the shape of the human femur, which has a substantially more prominent linea aspera than the porcine femur [31], and becomes more pronounced with age [34]. This longitudinal, posterior ridge can result in cerclage cable bridging, which could affect the net clamping force depending on the circumferential location of the fracture relative to the linea aspera. Thus, detailed measurements and statistical comparisons should be conducted on human femurs and would need to control for the linea aspera. Another important property in cerclage study design is the bone’s hardness since it is possible that changes in the bone surface at the cable/bone interface can result in tension loss apart from factors of the cerclage mechanical system.

The use of the linear viscoelastic model fitting of the measured relaxation data allows the calculation of estimates of the relaxation over times longer than the experiment, provided excellent fits of the data can be obtained. Here, fits with very high R^2^ values were obtained by using the starting value selection and iteration processes to determine the model parameters. The resulting curve fit parameters were used to estimate relaxation for up to 1 month. The estimates in Table 4 predict that in the tensile test experiments on the ss2.0 cables from Figure 4, essentially no additional relaxation occurs beyond 750 s, the time at which the experiment ended. For the long-duration results in the bone experiment (Figure 1 and Figure 7), both the cable tension and the clamping force cease relaxing after 1 day (Table 4). The additional reduction in cable tension after one day compared to that at the end of the test is negligible, whereas the bone clamping force is reduced by an additional 1.91%. The continued relaxation of the bone clamping force could be due to the cable’s interaction with the bone, which suggests that an additional retightening of the cable might eliminate this loss of clamping force.

Further investigation could involve in vivo testing. Continuous load measurements within a living specimen are challenging. Some reports [35,36] have demonstrated the use of radiopaque beads to assess the interval displacement using fluoroscopic imaging. However, the most clinically applicable testing strategy would involve a randomized control trial with treatment arms consisting of varying cable-tightening sequences and following patients postoperatively for radiographic and clinical outcomes.

## 5. Conclusions

The results of this study represent the amount of force reduction due to cable force relaxation and cable/bone interaction. As the focus of the study was cerclage cables, tension loss due to the process of crimping the crimp nut was not studied. The results here show that these cables relax at a rate sufficiently high enough that within the time of a typical surgery, the two-step loading process can greatly eliminate the initial loss of cable tension due to relaxation. An experiment on a pig femur confirmed the benefit of the two-step procedure based on the measurement of bone clamping force. The results also suggest that a very slow continuous loading procedure may further improve the retention of cable tension.

## Figures and Tables

**Table 1:** Average stress–strain properties (mean ± standard deviation) of cable specimens loaded to failure at 12 mm/min.

	Stainless Steel (d = 1.6 mm)	Stainless Steel (d = 2.0 mm)	Vitallium (d = 1.6 mm)	Vitallium (d = 2.0 mm)
Young’s Modulus (GPa)	61.45 ± 1.42	37.72 ± 1.49	61.99 ± 0.17	45.49 ± 8.22
Maximum Stress (MPa)	559.84 ± 1.62	825.46 ± 1.98	729.30 ± 17.58	798.26 ± 5.10
Failure Strain (%)	2.31 ± 0.14	5.38 ± 0.09	4.64 ± 0.73	6.03 ± 0.36

**Table 2:** Comparisons of loss of tension due to relaxation at 1200 s in Figure 3.

	*p*-Value
Stainless Steel (d = 1.6 mm) vs. Stainless Steel (d = 2.0 mm)	0.2496
Stainless Steel (d = 1.6 mm) vs. Vitallium (d = 1.6 mm)	0.2776
Stainless Steel (d = 1.6 mm) vs. Vitallium (d = 2.0 mm)	0.1551
Stainless Steel (2.0 mm) vs. Vitallium (d = 1.6 mm)	0.8562
Stainless Steel (2.0 mm) vs. Vitallium (d = 2.0 mm)	0.5051
Vitallium (d = 1.6 mm) vs. Vitallium (d = 2.0 mm)	0.3974

**Table 3:** Normalized cable tension at the end of the one-step and two-step procedures for the four trials along with the *p*-values for statistical comparison of the two procedures for each cable type. The lower part of the table compares the stainless-steel and vitallium cables.

	Final Normalized Tension						
Trial	1	2	3	4	Average	*p*-Value	Effect Size
Vitallium (d = 1.6 mm)							
One-step	0.9281	0.9293	0.9404	0.9328	0.9327	3.87 × 10^−5^	6.6
Two-step	0.9623	0.9655	0.9633	0.9671	0.9646		
Vitallium (d = 2.0 mm)							
One-step	0.9411	0.9368	0.9373	0.9281	0.9358	7.71 × 10^−6^	8.7
Two-step	0.9823	0.9765	0.9819	0.9781	0.9797		
Stainless Steel (d = 1.6 mm)							
One-step	0.9398	0.9360	0.9353	0.9221	0.9333	2.81 × 10^−4^	4.6
Two-step	0.9672	0.9675	0.9616	0.9730	0.9673		
Stainless Steel (d = 2.0 mm)							
One-step	0.9242	0.9152	0.9343	0.9373	0.9278	1.60 × 10^−4^	5.1
Two-step	0.9703	0.9701	0.9787	0.9729	0.9730		
Two-step	Stainless Steel (d = 1.6 mm)		vs.	Vitallium (d = 1.6 mm)		0.3212	0.7
	Stainless Steel (d = 2.0 mm)		vs.	Vitallium (d = 2.0 mm)		3.45 × 10^−2^	1.7

**Table 4:** Normalized relaxation forces after 750 s based on extrapolation of measured data using 5-parameter linear viscoelastic curve fits.

Normalized Force Values from Test Performed	750 (s)	1 Day	1 Week	1 Month
ss2.0 One-step cable tension (Figure 4)	0.932	0.931	0.931	0.931
ss2.0 Two-step cable tension (Figure 4)	0.973	0.972	0.972	0.972
Bone clamping force (Figure 7)	0.893	0.876	0.876	0.876
Cable tension (Figure 7)	0.870	0.867	0.867	0.867

## Data Availability

The original contributions presented in the study are included in the article. The data supporting the conclusions of this study are available from the corresponding author upon reasonable request.
